# Single-Molecule Long-Read Sequencing Reveals the Diversity of Full-Length Transcripts in Leaves of *Gnetum* (Gnetales)

**DOI:** 10.3390/ijms20246350

**Published:** 2019-12-17

**Authors:** Nan Deng, Chen Hou, Fengfeng Ma, Caixia Liu, Yuxin Tian

**Affiliations:** 1 Institute of Ecology, Hunan Academy of Forestry, Changsha 410004, China; idengnan@sina.com (N.D.); mafengfeng0403@126.com (F.M.); 2 Hunan Cili Forest Ecosystem State Research Station, Cili 417100, China; 3 School of life Sciences, Sun Yat-sen University, Guangzhou 510275, China; houchen1986@gmail.com

**Keywords:** *Gnetum*, full-length transcriptome, high throughout-put sequencing, *bHLH* gene, leaf

## Abstract

The limitations of RNA sequencing make it difficult to accurately predict alternative splicing (AS) and alternative polyadenylation (APA) events and long non-coding RNAs (lncRNAs), all of which reveal transcriptomic diversity and the complexity of gene regulation. *Gnetum*, a genus with ambiguous phylogenetic placement in seed plants, has a distinct stomatal structure and photosynthetic characteristics. In this study, a full-length transcriptome of *Gnetum luofuense* leaves at different developmental stages was sequenced with the latest PacBio Sequel platform. After correction by short reads generated by Illumina RNA-Seq, 80,496 full-length transcripts were obtained, of which 5269 reads were identified as isoforms of novel genes. Additionally, 1660 lncRNAs and 12,998 AS events were detected. In total, 5647 genes in the *G. luofuense* leaves had APA featured by at least one poly(A) site. Moreover, 67 and 30 genes from the *bHLH* gene family, which play an important role in stomatal development and photosynthesis, were identified from the *G. luofuense* genome and leaf transcripts, respectively. This leaf transcriptome supplements the reference genome of *G. luofuense*, and the AS events and lncRNAs detected provide valuable resources for future studies of investigating low photosynthetic capacity of *Gnetum*.

## 1. Introduction

*Gnetum* L., *Ephedra* L., and *Welwitschia* Hook.f. together form a monophyletic group—order Gnetales [1,2]. The phylogenetic relationship of the Gnetales with other seed plant groups has been a longstanding but controversial question of seed plant evolution [3,4,5,6,7]. On the basis of morphological and anatomical data, the Gnetales was inferred to be a sister group of angiosperms [2,8]. However, the most recent molecular phylogenies have placed the Gnetales as a sister group to Pinaceae or entire conifers [3,4,5,9]. Genus *Gnetum*, comprising approximately 40 species, are woody climbers, with a few exceptions being shrubs and trees [1,10,11]. Most of *Gnetum* species, such as the Asian lianoid *Gnetum*, are rich in bioactive compounds, which can be used for medicine [12,13,14].

*Gnetum* are characterized by their pinnate leaf veins, decussate leaves, and the presence of vessels in the stems [15]. The photosynthetic capacity of *Gnetum* was found to be lower than that of other seed plant groups, suggesting that this character acts as an intrinsic property and reflects a unique evolutionary history [1,16]. A very recent study uncovered the unique morphology of the *Gnetum* stomata [17], which to some extent explained the reasons for the low photosynthetic capacity, but underlying molecular mechanisms remain unknown. Wan et al. [3] proposed that cellulose synthase (*CesA*), cellulose synthase-like (*Csl*), and the WUSCHEL-related homeobox (*WOX*) family could play important roles of affecting gross morphology and leave development in *Gnetum*. These reported genes, however, are insufficient to explain the low photosynthetic capacity with regard to photosynthetic rate, stomatal conductance, and water transport capacity [1]. The *bHLH* (basic/helix-loop-helix) gene family has been reported to regulate stomatal development and photosynthesis, e.g., to initiate the asymmetrical division of protodermal cells, to terminate the meristematic stem cell identity, and to trigger flowering in response to blue light [18,19,20]. The *bHLH* family is characterized by bHLH (hidden Markov models, HMM accession: PF14215.6) and HLH (HMM accession: PF00010.26) domains [21]. It is the second-largest gene family in *Arabidopsis* (147 genes) and is also present in other plants (ranging from 1 to 553 genes). The discovery of the *bHLH* family promotes the understanding of molecular mechanisms that regulate low photosynthetic capacity in *Gnetum*.

Transcriptomes provide valuable gene resources for those gymnosperms whose reference genome is lacking. For those species that already have their whole genome sequenced, the transcriptomic data act as a good supplement to the genomic data, such as the detection of alternative splicing (AS) events and long non-coding RNAs (lncRNAs). Alternative splicing, which generates more than one transcript mediated by spliceosome (a large protein and RNA complex), is a major post-transcriptional regulatory mechanism in plants [22,23]. Alternative splicing has been proven to be involved in the regulation of growth, development, signal transduction, flowering, and responses to various environmental cues in plants [24,25,26,27]. Additionally, in recent years, lncRNAs have been reported as important regulators of gene expression, such as precursors of microRNAs (miRNAs) and miRNA target mimics [28,29].

At present, most transcriptomic data are generated using short reads sequencing, e.g., Illumina sequencing platforms. However, it is challenging to detect the presence of different isoforms, repetitive sequences, and transposable elements, because different transcripts which possess identical exons cannot be distinguished [30]. Thus, it is difficult to predict AS events and lncRNAs using short-read sequencing [31]. With the development of third-generation sequencing, the sequencing read length has grown rapidly, providing a more direct view of RNA molecules [32]. The third-generation sequencing technology overcomes the difficulties of short-read sequencing by generating the full-length sequence in a single molecule [33]. Currently, the average read length for single-molecule real-time (SMRT) sequencing is 12–15 kb, and the maximum length can reach 60 kb (PacBio Sequel platform). To date, SMRT sequencing has been applied to investigate full-length transcriptome in quite a few angiosperms species, e.g., sorghum [34], coffee [33], sugarcane [35], cotton [36], and the rubber tree [37], but barely been applied in gymnosperms.

This study performed the first SMRT transcriptome derived from *Gnetum luofuense* C.Y. Cheng leaves at different developmental stages using PacBio Sequel platform. To improve the sequence quality generated by PacBio Sequel platform, Illumina sequencing platforms were used to generate short reads. The annotation and structure analysis of the full-length transcripts are expected to improve the annotation of the reference genome of *G. luofuense* and to better understand the complexity of transcriptome in gymnosperms. Besides, the newly generated data can serve as the reference for differentially expressed analyses of *G. luofuense* leaves in further studies.

## 2. Results

### 2.1. Transcriptome from PacBio Sequel Sequencing

To reveal the complexity of the transcriptome in *G. luofuense* leaves, a pooled sample at different developmental stages was sequenced with the PacBio Sequel platform. In total, 9.98 GB of raw data was generated, made up of 3,689,825 subreads with an average length of 2705 bp and N50 of 3168 bp. A total of 185,089 circular consensus sequences (CCSs) were obtained, of which the full-length reads were 143,578 (Table 1). The full-length non-chimeric (flnc) reads were characterized by possessing complete 3′/5′ terminal primers and poly(A) tails. A total of 139,488 flnc reads were generated from *G. luofuense* leaf transcriptome, accounting for 75% of the CCSs (Table 1). After error correction, 80,496 polished consensus reads were obtained from Quiver, ranging from 167 bp to 14,735 bp (N50 of 3614 bp). The length distribution of the subreads, CCSs, and flnc reads was shown in Figure S1. To decrease PacBio Sequel sequencing errors, all PacBio sequenced transcripts were improved by the comparisons to the Illumina sequenced reads, whereupon the total numbers of nucleotides, mean lengths, and N50 were increased (Table 2). The lengths of *de novo* assembled unigenes were much shorter with the mean length of 972 bp than those generated from PacBio Sequel sequencing.

### 2.2. Transcript Structure Analysis

**Figure 1:**
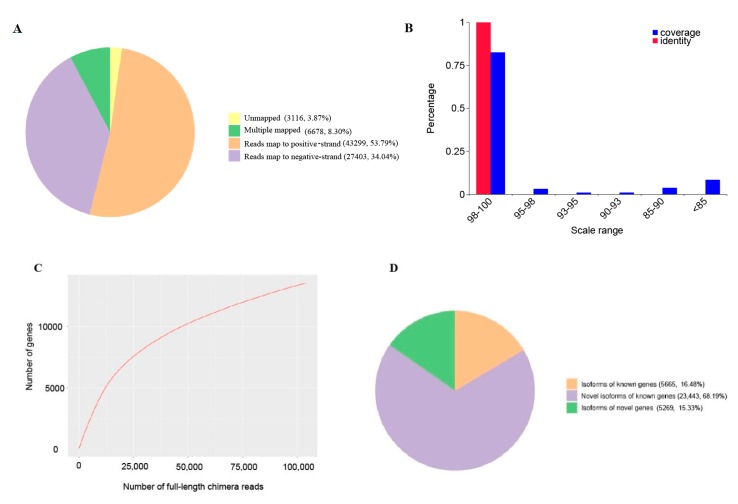
Results of mapping to reference genome. (**A**) Pie chart of mapped reads; (**B**) Range of mapping coverage and identity, x-axis represents the scale ranges, y-axis represents the percentages; (**C**) Saturation curve of consensus reads, x-axis represents numbers of full-length non-chimera (flnc) reads, y-axis represents numbers of genes; (**D**) Classification of flnc reads after mapping to the reference genome of *G. montanum* (= *G. luofuense*).

After error correction with the Illumina-sequenced data, 77,380 (96.31%) reads were mapped to the reference genome of *G. luofuense* using GMAP, leaving 3116 reads unmapped. Among the mapped reads, 6678 (8.30%) were mapped to multiple locations on the reference genome, 70,702 (87.83%) were uniquely mapped, and 43,299 (53.79%) and 27,403 (34.04%) were mapped to the positive strands and negative strands, respectively (Table 3, Figure 1A). Of the mapped reads, high-quality reads (i.e., with coverage and identity values over 98%) accounted for over 80% (Figure 1B), and the curve of the corrected isoform numbers reached a saturation level (Figure 1C). Overall, 93.01% of the de novo reads were successfully mapped to the reference genome (Table 3), where 5665 polished consensus reads (16.48%) were classified as isoforms from known genes, 23,443 (68.19%) were classified as novel isoforms from known genes, and 5269 (15.33%) were novel isoforms from novel genes (Figure 1D, File S1). The mean length and N50 of all isoforms were 3024 bp and 3394 bp, respectively. Figure S2A shows the density and number of the mapped reads on the reference genome of *G. montanum* (= *G. luofuense*). For 96.31% of all generated reads, scaffold498063 (the longest scaffold) had the most abundant mapped reads, whereas scaffold809851 and scaffold761035 had the lowest (Figure S2B).

### 2.3. Transcript Annotation and Classification

**Figure 2:**
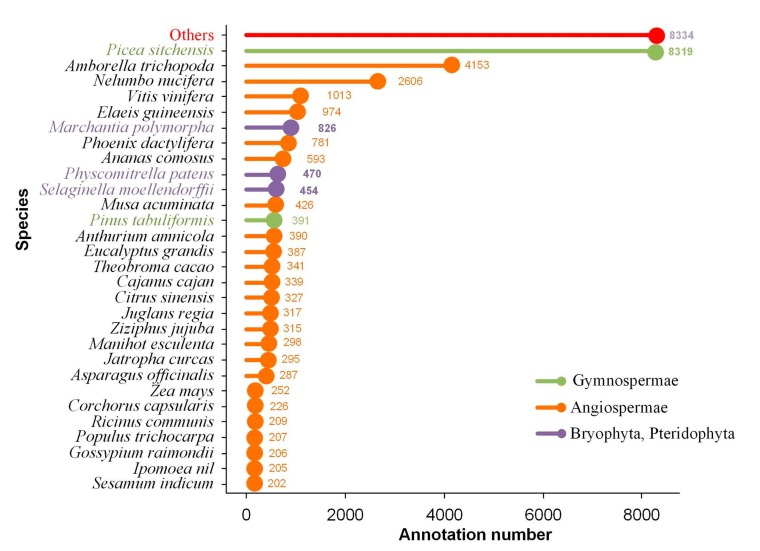
Nucleotide sequence (NR) (NCBI nonredundant protein sequences) homologous species distribution diagram of all genes and novel genes. Bars represent numbers of annotated consensus isoforms homologous to varied land plant species.

All the consensus isoforms were annotated by querying seven databases, i.e., gene ontology (GO), the Kyoto Encyclopedia of Genes and Genomes (KEGG), Eukaryotic Orthologous Groups/Clusters of Orthologous Groups (KOG/COG), Protein Family (Pfam), non-redundant protein sequences (NR), nucleotide sequence (NT), and Swiss-Prot. A total of 8980 isoforms had hits on all seven databases, and 34,667 had hits on at least one database. The NR database had the highest number of isoform annotations (34,170), followed by KEGG (33,813), whereas the NR database (15,467) had the lowest (Table 4). Furthermore, 5269 isoforms of novel genes were annotated against at least one database, and 116 isoforms had at least one hit in all databases. The NR database had the highest number of hits (3782). Additionally, 23,443 novel isoforms of known genes were searched against at least one database, and 6548 annotations of known genes had at least one significant hit on all databases, with the most being in NR database (22,167). All NR annotations were distributed among 256 species, where most of the consensus isoforms were homologous to those from *Picea sitchensis* (Bong.) Carrière (8319), *Amborella trichopoda* Baill. (4153), and *Nelumbo nucifera* Gaertn. (2606) (Figure 2).

**Figure 3:**
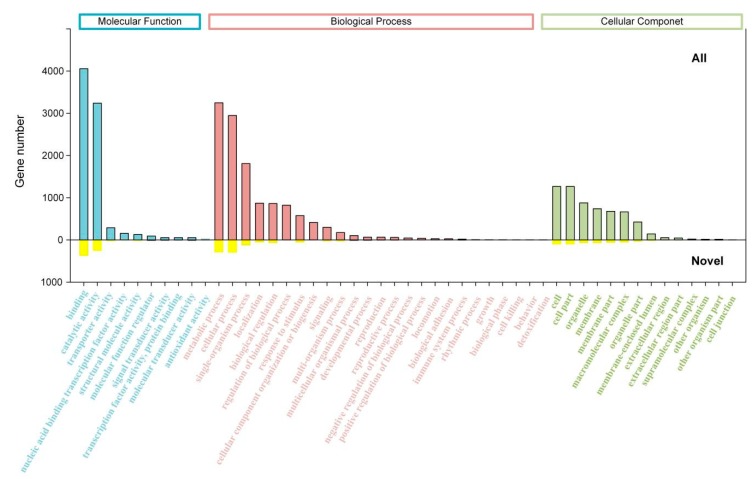
Functional annotation of full-length transcripts based on gene ontology (GO) categories. Bars represent the numbers of assignments proteins with BLASTx matches to each GO term. Upper bars represent GO annotation of all genes, and lower bars represent GO annotation of novel genes.

**Figure 4:**
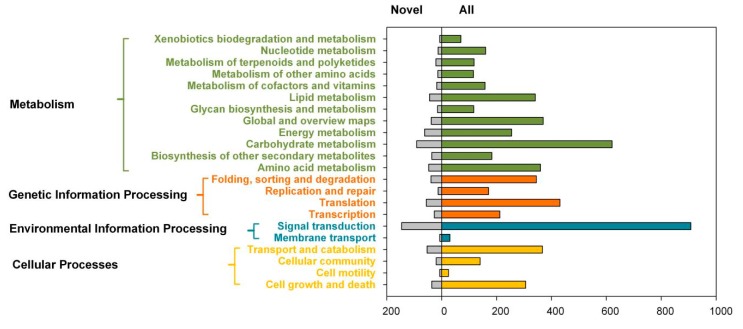
Functional annotation of full-length transcripts based on the Kyoto Encyclopedia of Genes and Genomes (KEGG) categories. Bars represent the numbers of assignments proteins with BLASTx matches to each KEGG term.

All the GO annotations were assigned to 51 GO categories (GO level 2, Figure 3). Among them, ‘binding’ (GO:0005488) represented the largest group (4056, 18.2%), followed by ‘metabolic process’ (GO:0008152) (3248, 14.6%) and ‘catalytic activity’ (3240, 14.5%). The 4006 novel genes were assigned to 43 GO categories, with the top three categories, i.e., ‘binding’, ‘catalytic activity’, and ‘metabolic process’. Besides, a total of 288 KEGG pathways were identified, of which novel genes were involved in 225 KEGG pathways. Among these KEGG pathways, the pathway ‘metabolism’ had the most abundant annotations of all genes and novel genes, followed by the orthologous term ‘signal transduction’ (908 for all genes and 146 for novel genes) (Figure 4).

### 2.4. Identification of LncRNAs and Fusion Genes

**Figure 5:**
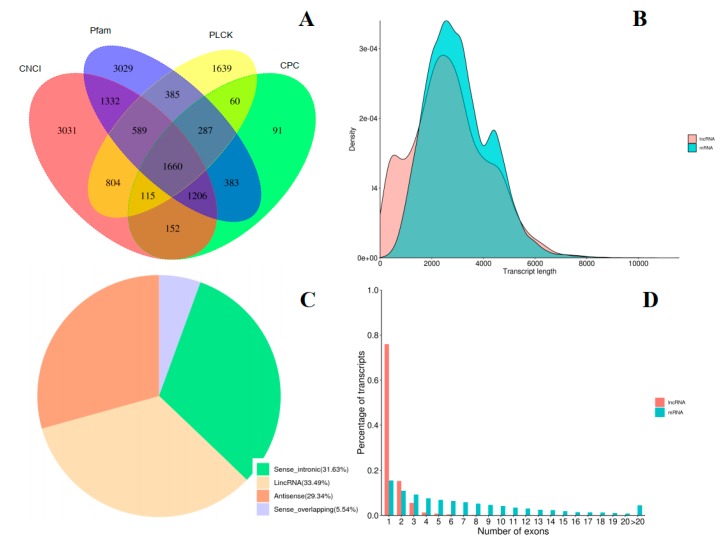
Analysis of long non-coding RNAs (lncRNAs) in *G. luofuense* leaves. (**A**) A Venn diagram of lncRNAs predicted by 4 methods; (**B**) The length density distribution of predicted lncRNAs and mRNAs in *G. luofuense* leaves, x-axis represents the length, y-axis represents the density; (**C**) Classification of predicted lncRNAs; (**D**) The distribution of exons numbers of mRNAs and predicted lncRNAs, x-axis represent numbers of exons, y-axis represent the percentages.

In total, 8890, 3955, 8871, and 5540 lncRNAs were identified by the Coding-Non-Coding-Index (CNCI), Coding Potential Calculator (CPC), Pfam, and PLEK databases, respectively (Figure 5A). Additionally, 1660 lncRNAs were identified by the four methods, with full lengths mainly ranging from 1000 bp to 5000 bp and lengths on average ranging from 203 bp to 8106 bp (File S2, Figure 5B). Among them, only 10 lncRNAs were identified as known transcripts whereas the others were identified as novel transcripts (Figure S3). All detected lncRNAs were subdivided into the following four types: lincRNAs (long intergenic non-coding RNAs, 556, 33.49%), sense intronic lncRNAs (525, 31.63%), antisense lncRNAs (487, 29.34%), and sense overlapping lncRNAs (92, 5.54%) (Figure 5C). Most of the identified lncRNAs had one or two exon(s), which was different from the exon distribution of regular mRNAs (Figure 5D). Additionally, 1174 fusion gene events were identified, of which 533 could be identified among the known genes, whereas the remaining fusion genes could not be linked to any known genes, probably there are still many undetected genes in the reference genome of *G. luofuense* (Figure S3).

### 2.5. Transcription Factor, Alternative Polyadenylation, and Alternative Splicing Analyses

**Figure 6:**
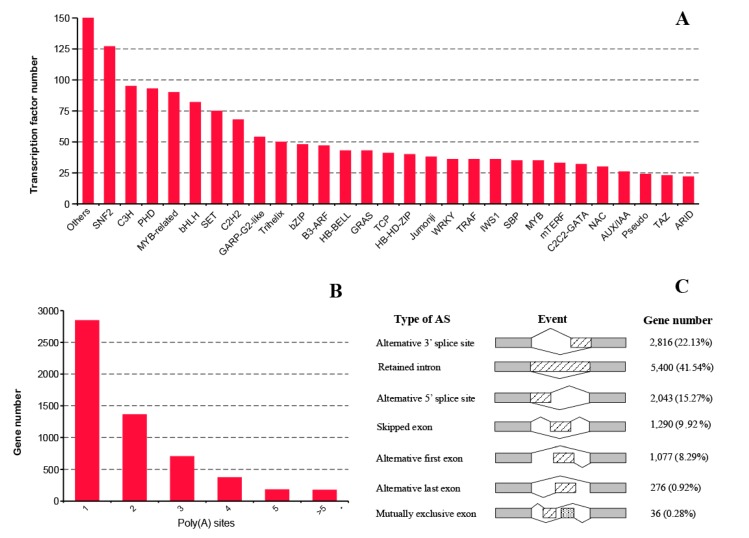
(**A**) Number of transcript factors identified in the present study; (**B**) Varied types of alternative polyadenylation and corresponding gene numbers; (**C**) A schematic graph that illustrates different types of alternative splicing.

A total of 1974 transcription factors (TFs) were identified by iTAK (Ithaca, NY, USA), of which 1824 TFs were annotated to 82 families (Figure 6A). Sucrose nonfermenting 2 (SNF2) accounted for the greatest proportion in the known TF families, followed by coumarate-3-hydroxylase (C3H) (Figure 6A). In addition, 5647 genes in the *Gnetum* leaves were found to have at least one supported poly(A) site (Figure 6B). The transcripts with one poly(A) site were in the highest proportion (50.40%), followed by genes with two poly(A) sites (24.14%). The results of APA analysis were presented in File S3.

A total of 12,998 AS events (assigned to 4459 genes) were detected, comprising 5,400 (41.54%) retained introns, 2,876 (22.13%) alternative 3′ splice sites, 2043 (15.27%) alternative 5′ splice sites, 1290 (9.92%) skipped exons, 1077 (8.29%) alternative first exons, 276 (2.12%) alternative last exons, and 36 (0.28%) mutually exclusive exons (Figure 6C). Additionally, an alternative 3′ splice site was detected in all the transcripts from novel genes.

### 2.6. Phylogenetic Analysis of bHLH Genes in G. luofuense

**Figure 7:**
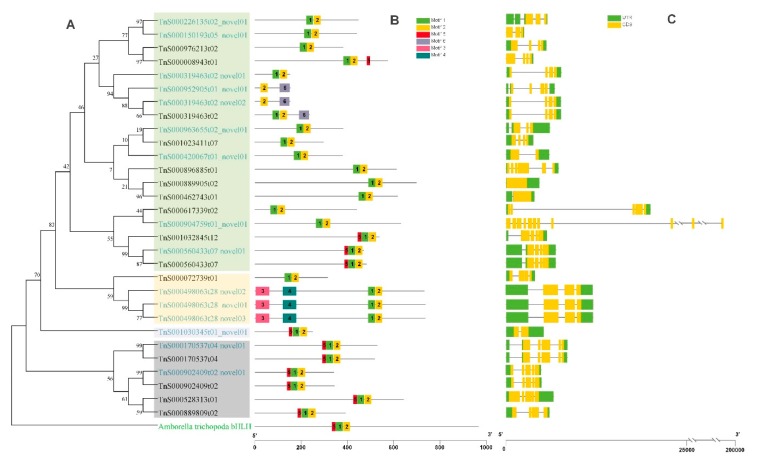
Gene structure, motif, and phylogenetic analysis of 30 *bHLH* genes. (**A**) A phylogenetic tree of bHLH transcription factors using a neighbor-joining method; (**B**) Conserved motifs in *bHLH* genes were marked in different colors; (**C**) Gene structures of *bHLH* genes in *Gnetum*.

A total of 67 *bHLH* genes were detected by searching against the reference genome of *G. luofuense* with regard to the bHLH or HLH domain. The information of the *bHLH* gene family members in *Gnetum* was presented in File S4. Besides the bHLH and HLH domains, the KIX_2 (HMM accession: PF16987.5), AAA_33 (HMM accession: PF13671.6), Macro (HMM accession: PF01661.21), DcpS_C (HMM accession: PF11969.8), and zf-C2HE (HMM accession: PF16278.5) domains were also identified in the *Gnetum bHLH* gene family. A rooted neighbor-joining tree of the 67 bHLH proteins from *G. luofuense* was shown in Figure S4. A further concentrated phylogeny of 30 *bHLH* genes (including 15 novel genes) detected in the full-length transcriptome was reconstructed (Figure 7), four subfamilies were separated from one another, however some deep nodes had low statistical support. In addition, almost all the detected bHLH proteins contained conserved motifs 1 and 2 and the two motifs were close to each other, suggesting the presence of the two conserved domains. In contrast, motif 5 had the second widest distribution, whereas motif 3 and 4 were only found in one gene (*TnS0004498063t28*).

## 3. Discussion

**Figure 8:**
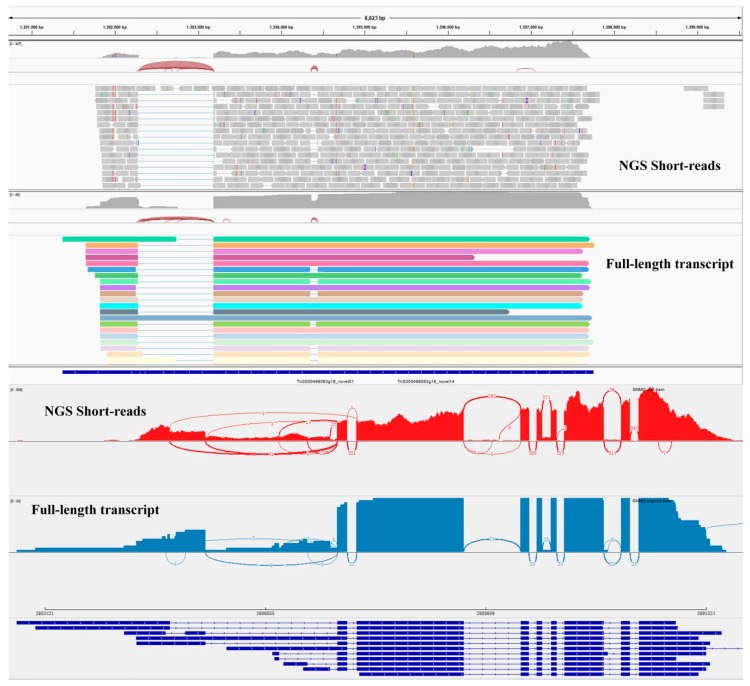
Different transcript isoforms of one *bHLH* gene detected in the leaf transcriptome mapped to the reference genome of *G. luofuense*.

In the present study, the third-generation sequencing technology was applied to generate full-length transcriptome of *G. luofuense* leaves. 80,496 polished consensus reads were obtained with an average length of 3223 bp. The PacBio Sequel platforms gained considerably longer transcripts than those generated in Illumina platforms, providing a superior overview of the *Gnetum* transcriptome. After mapping the reads of PacBio and Illumina sequencing against the reference genome, respectively, we were able to visualize the coverages and AS events (Figure 8). Fifteen isoforms with a high coverage were detected (with lengths of over 5000 bp) from the *bHLH* gene family, providing a huge advantage over the short reads which needed assembly. PacBio Sequel sequencing is subject to higher rates of errors [38], and a previous study reported error rates could reach to 11–14% [39]. Therefore, in this study, we applied Illumina-sequenced data to correct errors generated from Pacbio Sequel sequencing and made the complete assessment of our assembled full-length transcriptome.

LncRNAs, which are operationally defined as RNA genes with a length of over 200 bp, have elusive functions as they are not responsible for protein coding [40]. Although lncRNAs play an important role in gene regulation in plants [28], their numbers, characteristics, and genetic patterns remain unclear [40]. So far, lncRNAs have been identified in many angiosperms, such as *A. thaliana* [41], *Oryza sativa* [42], and *Morus notabilis* [43], very few lncRNAs have been identified in gymnosperms. The lncRNAs in plants have been known to participate in root, stem, and leaf development [44]. For example, lncRNA-HID1 acted as a factor of promoting photomorphogenesis in light [45]. Another case shows that the overexpression of lncRNA-npc48 resulted in *A. thaliana* leaf serration and delaying flowering time [46]. Moreover, lncRNA (TWISTED LEAF) played an important *cis-*regulatory role to regulate the expression of gene *OsMYB60* during leaf development [47]. Function prediction of lncRNAs is challenging owing to the lack of the homology between closely related species [48]. In this study, we used four methods to identify lncRNAs in *Gnetum* leaves, whereupon 1660 transcript sequences were identified as putative lncRNAs and most of them contained one exon or two exons. These results provide a base to better understand the varied roles of lncRNAs in gymnosperms.

A large number (5269, 15.33% of total transcripts) of isoforms from novel genes were detected but with a lack of annotation. The newly detected genes are deemed to enrich the knowledge of the reference genome of *G. luofuense*, although our sampling for long-read transcriptome sequencing is restricted to leaves. The predicted novel genes and novel isoforms may provide valuable opportunities to figure out the gene functions involved in leaf development of *G. luofuense*. In addition, 23,443 novel isoforms of known genes were detected, the result suggests that AS events frequently occur in *Gnetum*. It is more confident to predict AS events based on full-length transcriptome than those assembled by short reads because of the complexity of the AS mechanism in eukaryotic cells [35]. Our results indicate that the retention of introns was the main AS type found in the *Gnetum* leaves, congruent with sugarcane [35] and strawberry [49] as reported in previous studies.

Members of *bHLH* gene family diverge largely between gymnosperms and angiosperms, the later possesses an even larger range of gene numbers from 150 to 553. To date, *Picea abies* is known to have the most abundant *bHLH* genes (107) among the gymnosperms, followed by *G. montanum* (64). In this study, 30 *bHLH* genes including 15 novel genes were identified from the leaf transcriptome data, but the deep divergence of the phylogenetic tree received poor support. This result is also found in other organisms, it is probably because the conserved domains of *bHLH* genes are short and the remaining regions are highly divergent [50,51]. According to the neighbor-joining tree, the *bHLH* genes identified in *G. luofuense* leaves were resolved to four clades, but phylogenetic relationships between the different clades were vague because of the poor support. The *bHLH* genes may have recent common origins derived from several genomic duplication events, and the sequence divergence outside the domains may have experienced extensive shuffling events afterward [52].

## 4. Materials and Methods

### 4.1. Samples Selection and RNA Extraction

Leaves of *G. luofuense* were collected from a bamboo garden at Sun Yat-sen University, Guangzhou, China on 8–9 May 2018. To obtain a good representation of the *Gnetum* transcriptome, leaves at different developmental stages (from young to old) were collected to cover the entire transcriptome. All the leaf samples for RNA isolation were stored in RNA protection reagent (Qiagen, Hilden, Germany) at −20 ℃.

The total RNA of the sample was extracted using a TRIzol kit (Invitrogen, Carlsbad, CA, USA) according to a previously described two-step protocol [53]. The RNA quality, integrity, and quantity were determined using the following four methods: 1) the RNA samples were examined by agarose gel electrophoreses to verify whether the RNA had degraded or not; 2) the RNA purity was tested using a NanoDrop spectrophotometer (ThermoFisher Scientific, Wilmington, DE, USA); 3) the RNA was quantified using Qubit; and 4) the RNA integrity was examined using the Agilent Bioanalyzer 2100 system (Beijing, China). The concentration of total RNA extracted was over 300 μg/μL, with a mass yield of at least 5 μg. The OD_260/280_ value for each sample was 2.0–2.2, and the OD_260/230_ was 1.8–2.1. For PacBio Sequel sequencing, two rounds of sample pooling were carried out. Firstly, we combined the leaf samples of *G. luofuense* at three developmental stages for cDNA library construction. Secondly, leave samples were pooled for the Illumina RNA-Seq, and the methods of RNA extraction, sequencing, and assembly referred to our previous study [13].

### 4.2. Sample Preparation and PacBio Sequel Sequencing

PacBio Sequel sequencing was performed with the Clontech SMARTer PCR cDNA Synthesis Kit and BluePippin Size-Selection System (Sage Science, Beverly, MA, USA). One pooled sample was subjected to purification and size selection according to the PacBio Sequel protocol: (1) The cDNA was synthesized using a Clontech SMARTer PCR cDNA Synthesis kit (Clontech, Takara Bio Inc., Shiga, Japan) and amplified using a KAPA HIFI PCR kit (Kapa Biosystems, Boston, MA, USA); (2) the cDNA was purified using a QIAquick PCR Purification kit (Qiagen, Hilden, Germany) and then precipitated and normalized with a Trimmer-2 cDNA Normalization kit (Evrogen, Moscow, Russia). Full-length cDNA damage/terminal repair and SMRTbell template preparation were then carried out. The size-selection protocol was applied because the smaller cDNAs were abundant and should be, therefore, preferentially sequenced. The RNA-seq datasets generated are available from the NCBI Sequence Read Archive database (SRA) under BioProject number accession PRJNA572572.

### 4.3. PacBio Sequencing Data Processing

The sequencing data were processed using PacBio SMRTlink (5.1) software. Firstly, circular consensus sequences (CCSs) were generated from the subreads.bam file adapters (effective insert of single molecules), with the following parameter settings: min_length, 200; max_drop_fraction, 0.8; no_polish, true; min_zscore, −9999; min_passes, 1; min_predicted_accuracy, 0.8; and max_length, 18,000. All the CCSs were classified as full-length reads and non-full-length reads with the following two parameters: ignoring poly(A), false and minSeqLength, 200. The full-length reads were identified considering the presence of 5′ adapter sequences, 3′ adapter sequences, and poly(A) tails. The non-full-length and full-length isoforms were clustered to generate the consensus using an isoform-level clustering algorithm. To obtain full-length polished consensus sequences, Quiver software was used to correct PacBio sequencing errors with the following parameters: hq_quiver_min_accuracy, 0.99; bin_by_primer, false; bin_size_kb, 1; qv_trim_5p, 100; and qv_trim_3p, 30. To reduce the error rates in transcript prediction and ensure transcriptome completeness, a homology search against the Pfam database was conducted. Additional nucleotide errors in consensus reads were corrected using the Illumina RNA-Seq data with the software LoRDEC (Helsinki, Finland) [54]. Additionally, consensus sequences were mapped to the reference genome of *G. montanum* (= *G. luofuense*) [3] using GMAP [55] with the following parameter settings: -f samse -n 0; min-trimmed-coverage, 0.85, and min-identity, 0.9.

### 4.4. Structure Analysis and Annotation

Gene structure analysis was performed using the TAPIS pipeline, the GMAP output files in bam and gff/gtf formats were used for gene and transcript determination, read clusters that overlapped non-annotated genes were classified as novel genes, and AS and alternative polyadenylation (APA) events were analyzed. Additionally, fusion transcripts were determined as the transcripts mapping to two or more long-distance-range genes and were validated by at least two Illumina reads. The ANGEL pipeline, a long-read implementation of ANGLE [56], was used to determine the protein-coding sequences from the cDNAs. The transcription factors (TFs) were predicted using software iTAK [24].

LncRNAs, which do not encode proteins, are a type of RNA with a length above 200 nt. In this study, lncRNAs were detected using the following databases: Coding-Non-Coding-Index (CNCI) [57], Coding Potential Calculator (CPC) [58], PLEK [59], and Pfam. CNCI, which was set to default parameters in this study, profiles adjoining nucleotide triplets to distinguish protein-coding from non-coding sequences. CPC was used to assess the integrity of the open reading frame in a transcript, whereas Pfam Scan was used to identify known protein family domains with the parameters (-E 0.001 and -domE 0.001). Any transcript with a Pfam hit was excluded. Default parameters were used for the Pfam searches. The NCBI’s eukaryotic protein database was used to clarify the coding and non-coding transcripts with *e*-value < 10^−10^.

Gene Ontology (GO) enrichment analysis of the full-length transcriptome was conducted using the GOseq package [60] implemented in R (R core team 2018). All full-length transcripts were submitted to the Kyoto Encyclopedia of Genes and Genomes (KEGG) database (http://www.genome.jp/kegg/) to obtain KEGG Orthology assignments. In addition, assembled transcripts were searched against the NCBI’s non-redundant protein sequences (NR), nucleotide sequence (NT) databases, the Swiss-Prot (a manually annotated and reviewed protein sequence database) database with *e*-value < 10^−5^, and the Pfam (database of protein families and domains) database with *e*-value < 10^−10^. KOBAS software [61] was used to test the statistical enrichment of the transcriptome in the KEGG pathways. Picard tools and SAM tools [62] were, respectively, used to mark and sort duplicated reads and to re-order the alignment results for each sample and to detect single nucleotide polymorphism.

### 4.5. Phylogenetic Analysis

A total of 225 bHLH protein sequences in *Arabidopsis thaliana* were downloaded from PlantTFDB (http://planttfdb.cbi.pku.edu.cn/) and were searched for homology against the reference genome of *G. montanum* (= *G. luofuense*) [3] and the full-length transcriptome of *G. luofuense* generated in this study. The domain architectures of putative bHLH transcription factors were annotated using the Pfam HMMs: bHLH (PF14215.6) and HLH (PF00010.26). All bHLH proteins were aligned using ClustalW, and neighbor-joining trees were constructed with 1000 bootstrap iteration using software MEGA (Hachioji, Japan) [63]. The motif was predicted using MEME version 5.0.5 (Reno, NV, USA), where the number of motifs was set as 6 and the motif site distribution was set as 0 or 1 occurrence per sequence.

## 5. Conclusions

The present study generated 80,496 full-length transcripts with a long N50 length to reveal the complexity of full-length transcriptome during leaf development of *G. luofuense*. To overcome the defects of PacBio Sequel sequencing, Illumina sequenced data of the same sample were applied to improve the quality of the consensus reads. It is noteworthy that a large number of novel genes and novel isoforms were detected in the present study, improving our understanding of the reference genome of *G. luofuense*. The full-length transcriptome could serve as a reference to further detect differentially expressed genes and isoforms and to seek for genes of interest in order to uncover gene functions during leaf development of *G. luofuense.* Moreover, the AS events and lncRNAs detected in the leaf transcriptome also provide additional resources for the study of the low photosynthetic characteristic of this genus.

## Figures and Tables

**Table 1:** Summary of PacBio sequencing data in *Gnetum. luofuense.*

Terms	Amount
Subreads bases	9.98 G
Number of subreads	3,689,825
Average length of subreads	2750 bp
N50 of subreads	3168 bp
Number of CCSs	185,089
Number of sequences with 5′ terminal primers	167,590
Number of sequences with 3′ terminal primers	166,862
Number of sequences with poly(A) tails	156,554
Number of full-length sequences	143,578
Number of full-length non-chimeric (flnc) reads	139,488
Average length of flnc reads	3065 bp
Percentage of flnc reads	75%
Number of polished consensus reads	80,496
Minimum length of consensus reads	167 bp
Maximum length of consensus reads	14,735 bp
Average length of consensus reads	3223 bp
N50 of consensus reads	3614 bp

**Table 2:** Summary of data correction of PacBio sequencing data in comparison with Illumina sequenced short reads.

Type	Before Correction (Pacbio Sequel)	After Correction (Pacbio Sequel)	Short Reads (Illumina)
Total nucleotide	259,381,401	260,514,867	44,269,498
Total sequence	80,496	80,496	45,566
Mean length	3223 bp	3237 bp	972 bp
Minimum length	167 bp	167 bp	201 bp
Maximum length	14,735 bp	14,734 bp	12,325 bp
N50	3614 bp	3629 bp	2030 bp
N90	2102 bp	2102 bp	323 bp

**Table 3:** Comparisons of Pacbio and Illumina sequenced data with regard to read mapping.

Terms	PacBio Sequenced Data		Illumina Sequenced Data	
	Number of Reads	Percentage	Number of Reads	Percentage
Total mapped	77,380	96.13%	54,289,038	93.01%
Unmapped	3116	3.87%	4,079,995	6.99%
Multiple mapped	6678	8.30%	1,691,837	2.90%
Uniquely mapped	70,702	87.83%	52,597,201	90.11%
Uniquely mappedto positive strands	43,299	53.79%	26,287,949	45.04%
Uniquely mappedto negative strands	27,403	34.04%	26,309,252	45.07%

**Table 4:** Numbers of annotated consensus isoforms by the search against different databases.

Database	Total Number of Annotated Isoforms	Number of Novel Genes	Number of Novel Isoforms of Known Genes	Number of Isoforms of Known Genes
NR	34,170	3782	22,167	8221
SwissProt	28,954	2741	19,023	7190
KEGG	33,813	3659	22,005	8149
KOG	21,723	1925	14,658	5140
GO	22,297	1047	15,180	6070
NT	15,467	936	10,680	3851
Pfam	22,297	1047	15,180	6070
In all databases	8980	116	6548	8281
At least in one database	34,667	5269	23,443	2316

## Supplementary Materials

Supplementary materials can be found at https://www.mdpi.com/1422-0067/20/24/6350/s1.
