# Supplementary material for: Single-Molecule Long-Read Sequencing Reveals the Diversity of Full-Length Transcripts in Leaves of Gnetum (Gnetales)
Source: Int J Mol Sci. 2019 Dec 17;20(24):6350. doi: 10.3390/ijms20246350 (PMC6941099; doi:10.3390/ijms20246350)
Supplement: Supplementary file 1 [file ijms-20-06350-s001.zip › ijms-627358-supplementary figures.pdf]

Supplementary Figures

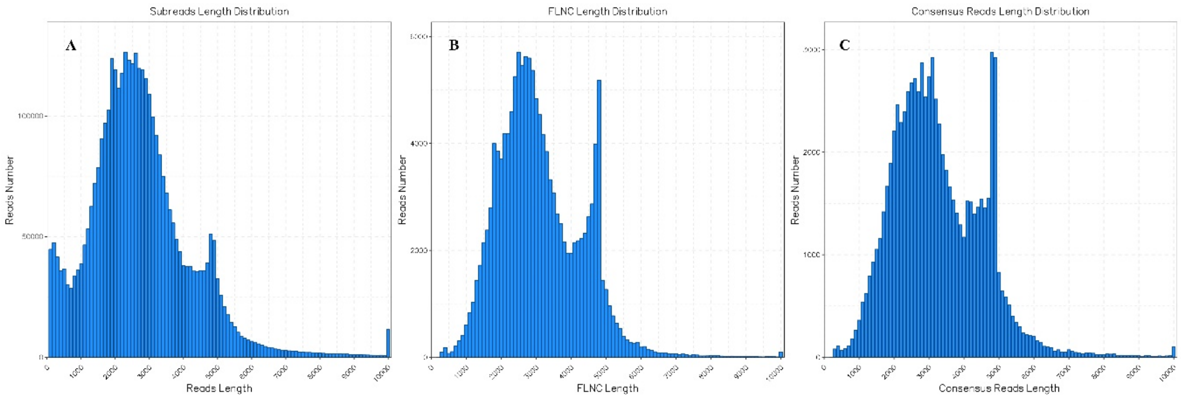

Figure S1. Length distribution of subreads (**A**), flnc (**B**) and consensus reads (**C**). x-axis represents the length, y-axis represents read numbers.

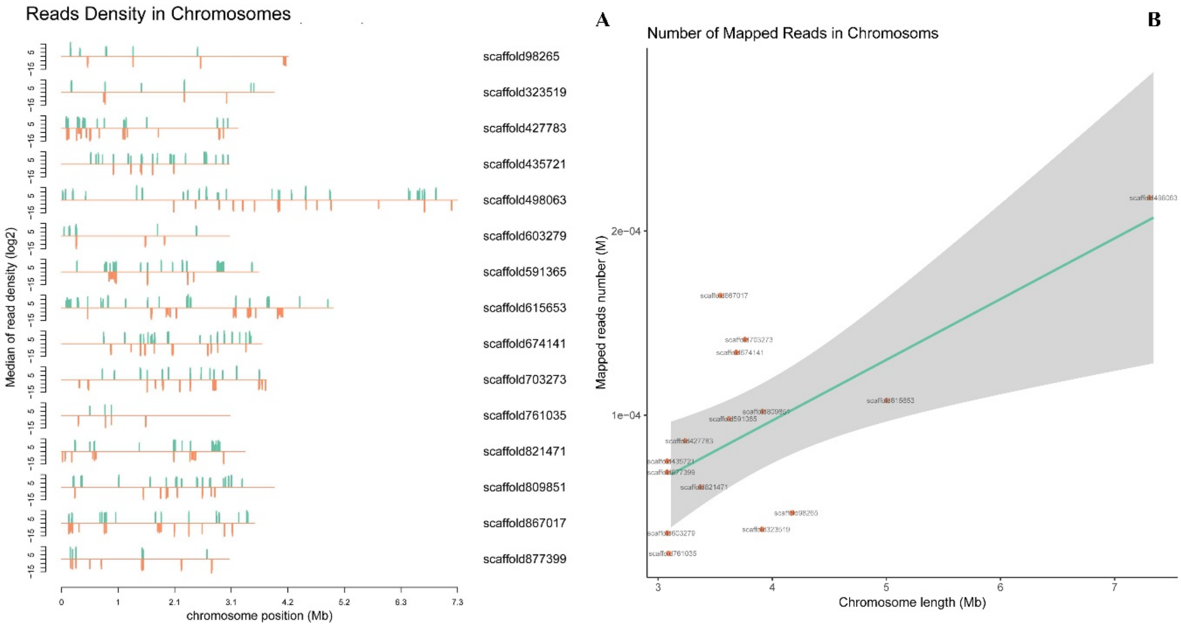

Figure S2. Number and density of mapped reads in several scaffolds. (**A**) Mapped reads density in scaffold, x-axis represents the scaffold position (Mb), y-axis represents the median of read density (log2), green and red vertical lines represent the positive strands and negative strands, respectively; (**B**) Number of mapped reads in several scaffolds, x-axis represents the scaffold position (Mb), y-axis represents numbers of mapped reads, the grey area shows the a confidence interval of 95%.

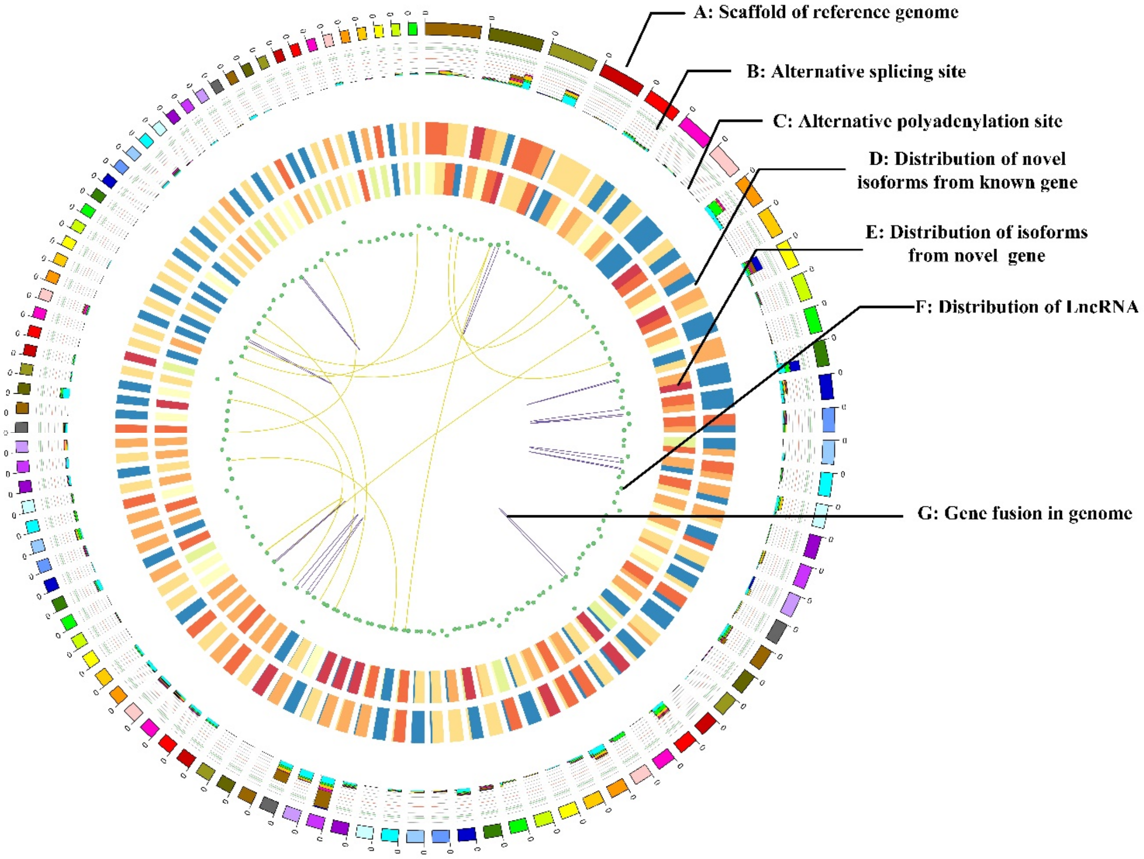

Figure S3. Visualization of Sequel sequencing data at the genome-wide level. (**A**) Scaffolds of the reference genome of *G. luofuense*, (**B**) Alternative splicing sites on the reference genome, different color represent varied types of alternative splicing events; (**C**) Alternative polyadenylation sites; (**D**) Distribution of novel isoforms from known genes, the red color represents the high density; (**E**) Distribution of isoforms from novel genes, the red color represents the high density; (**F**) Distribution of lncRNAs; (**G**) Gene fusion events detected on the reference genome.

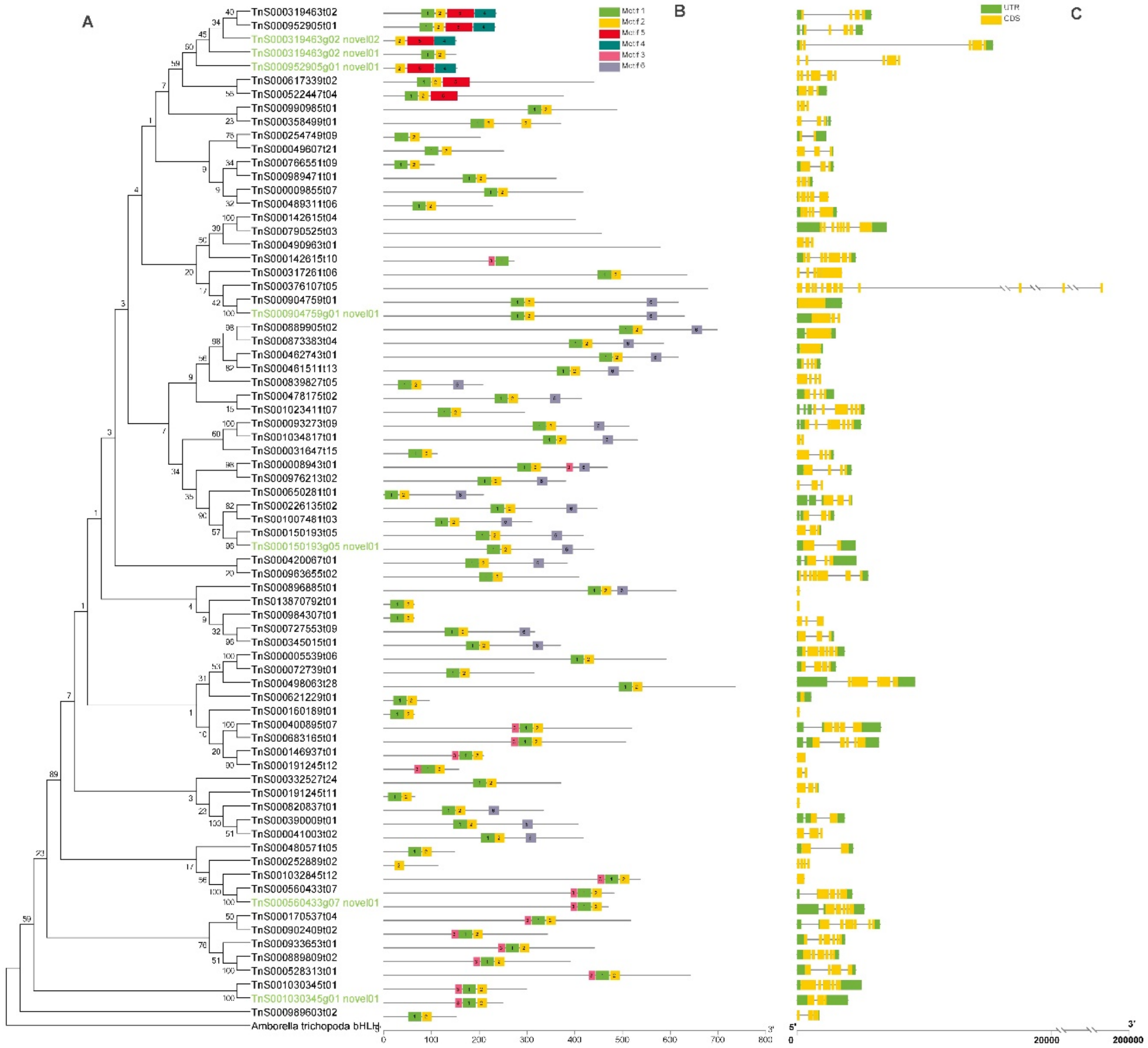

Figure S4. Gene structure, motif and phylogenetic analysis of *bHLH* genes. (**A**) The phylogenetic tree of the proteins constructed using the neighbour-joining method; (**B**) conserved motifs in *bHLH* gene; (**C**) Gene structures of *bHLH* genes.

Supplementary Files

File S1. List of isoforms from novel genes in *Gnetum* leaves.

File S2. List of the predicted lncRNAs.

File S3. Results of poly(A) prediction.

File S4. List of *bHLH* gene family identified in *Gnetum* genome and leaf transcriptome.
